# Thermodynamics and historical relevance of a jetting thermometer made of Chinese zisha ceramic

**DOI:** 10.1038/srep28609

**Published:** 2016-07-19

**Authors:** Vincent Lee, Daniel Attinger

**Affiliations:** 1 Mechanical Engineering Department, Iowa State University, Ames IA 50010, USA; 2 Mechanical Engineering Department, Georgia Institute of Technology, Atlanta, GA 30332, USA.

## Abstract

Following a recent trend of scientific studies on artwork, we study here the thermodynamics of a thermometer made of zisha ceramic, related to the Chinese tea culture. The thermometer represents a boy who “urinates” shortly after hot water is poured onto his head. Long jetting distance is said to indicate that the water temperature is hot enough to brew tea. Here, a thermodynamic model describes the jetting phenomenon of that *pee-pee boy*. The study demonstrates how thermal expansion of an interior air pocket causes jetting. A *thermodynamic potential* is shown to define maximum jetting velocity. Seven optimization criteria to maximize jetting distance are provided, including two dimensionless numbers. Predicted jetting distances, jet durations, and temperatures agree very well with infrared and optical measurements. Specifically, the study confirms that jetting distances are sensitive enough to measure water temperature in the context of tea brewing. Optimization results show that longer jets are produced by large individuals, with low body mass index, with a boyhood of medium size inclined at an angle π/4. The study ends by considering the possibility that ceramic jetting artifacts like the pee-pee boy might have been the first thermometers known to mankind, before Galileo Galilei’s thermoscope.

Since the beginning of this millennium, there has been an exponential growth of scientific studies on artwork. For example, a search on the scientific database Web of Science for publications matching both “scientific” and “artwork” topics shows more than 140 studies per year in 2014, much higher than the average of 5 studies per year in the 1990s. Scientific studies have been conducted on artwork for determining their production date and materials^1^,^2^,^3^,^4^,^5^,^6^, for quantitatively characterizing their style^7^,^8^,^9^, significance^10^, creativity^11^,^12^ and even beauty^13^, and for preservation or maintenance purposes^14^,^15^,^16^,^17^,^18^,^19^. A wide range of measurement techniques have been used such as electromagnetic measurements^4^,^19^ from X-rays^20^,^21^ to infrared wavelengths^1^,^22^, Raman spectroscopy^4^,^23^,^24^,^25^, mass spectrometry^26^,^27^,^28^, image analysis^8^,^9^, rheology measurements^7^, and biochemistry assays^5^. Preservation techniques have recently used laser processing^17^,^29^, as well as innovative soft and nano-materials^15^,^30^.

Here the object of study is a hollow and boy-shaped ceramic artifact shown in Fig. 1, which belongs to a class of ornamental ceramic objects associated with tea brewing^31^, and called tea pets. Often made of reddish zisha ceramic, a clay found in the Yixing county of Jiangsu province with over nine percent of iron, tea pets come shaped in the form of natural objects such as trees, vegetables, and animals^31^,^32^. A function of these zisha tea pets is to ‘*surprise, intrigue, provoke a smile, or excite erudition*’^33^. Adepts talk about ‘raising’ the tea pet, in Chinese ‘*yanghu*’. Personal communication with Geoffrey Gowlland, a postdoctoral fellow at the Museum of Cultural History in Olso, Norway, suggests that the concept of tea pets comes from a play on those meanings of the word *‘yang’*, which means raising or feeding animals, as well as creating a deeply pleasant and shiny patina by repeatedly pouring tea on zisha potteries, so that the tea tannins penetrate the porous surface of the unglazed ceramic. The Chinese culture of tea is comparable in importance and sophistication to that of fine wines in France^34^. Zisha potteries have been produced in China since the Song dynasty (960–1260), traditionally in the Yixing county of Jiangsu province. Zisha pots were the first type of Chinese ceramics to be exported to Europe^31^.

The tea pet in Fig. 1b is colloquially known as a pee-pee boys for a reason as shown in Fig. 1; shortly after a stream of hot water is poured onto the head of the pee pee boy, its ceramic penis produces a water jet. Vendors of pee-pee boys and Wikipedia^35^ suggest that a long, powerful jet indicates that water is hot enough to brew tea, that is, the pee-pee boy can serve as a *thermometer*. Certainly, the water temperature and infusion time significantly affect the quality of tea^36^. Personal communication with Geoffrey Gowlland suggest that other tea pets that possibly operate with the same mechanisms include a water-spewing frog, the Tang Dynasty toad in Fig. 1. Thermodynamic machines such as the pee-pee boy create surprise by ‘*magically’* transforming heat into mechanical work. Examples include the acoustical heat engine^37^ or the dunking duck^38^. The latter is a hollow device which oscillates quasi-perpetually between a vertical and a drinking position by virtue of successive evaporation, condensation, and internal liquid displacement.

In this manuscript, we test and confirm the hypothesis that the jetting process is produced by the thermally-driven expansion of an air pocket in the pee-pee boy’s head, which pushes the internal liquid into a jet. The ability to measure temperature based on the maximum length of the jet is investigated and discussed. A theoretical model is constructed to describe the pee-pee boy as a thermometer and a heat engine. We numerically solve the equations of thermodynamics, heat transfer and ballistic, with quantitative parameters obtained from measurements. The evolution of the temperatures, the internal pressure, as well as the jetting process, is described and compared to measurements. Dimensionless numbers are also defined, which guide the design of tea pets to reach jetting distances close to the identified thermodynamic limit.

## Methods

Pee-pee boys come with various demeanors and shapes, as shown in Fig. 2. They have approximately the height of a tea cup (~7 cm), with a disproportionately large head. Six pee-pee boys have been used for this study, obtained from a popular shopping website. No significant differences in color, size, weight and performance were found between these tea pets and two other initially obtained from a large zisha pottery store near Beijing.

### Static characterization

Sections of three pee-pee boys were cut with a diamond saw, and the thickness of their ceramic envelope was measured with a mechanical caliper (0.01 mm uncertainty). A miniature circular saw blade was used to expose smaller features, such as the cross-section of the pee hole, see Fig. 2. The equivalent diameter of the disk-shaped pee holes was estimated as *d* = 4*A*/*P*, where *A* is the cross-sectional area, and *P* is the perimeter of the hole. This expression used in hydraulics is called the hydraulic diameter, with the factor four ensuring that *d* of a perfect disk equals its natural diameter. The internal volume was determined with a precision balance (0.1 g uncertainty) by comparing the weight of a water-filled device with that of an empty one. The volume of jetted water was measured by weighing the tea pet before and after jetting.

### Jetting experiments

To ensure reliable jetting, the pee-pee boy needs to be partially filled with water, and brought to room temperature, as follows. First, two large beakers (500 mL) and one small beaker (80 mL) are filled with water at room temperature. One large beaker is put to boil in a microwave oven. The pee-pee boy is first immersed in the hot beaker to evacuate air by expansion and bubbling from the pee hole. The pee-pee boy is then immediately immersed in the beaker at room temperature for another minute. This process fills the pee-pee boy, by thermally contracting its internal air pocket, and cools down the pee-pee boy to the ambient reference temperature.

**Figure 1:**
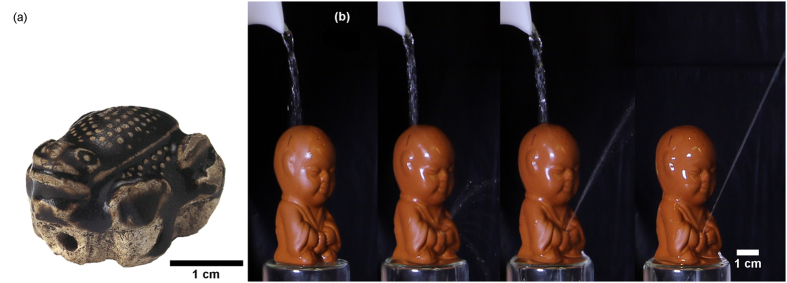
Ceramic artwork offer a testimony of Chinese culture, as with this Tang dynasty (618–906 CE) frog-shaped whistle (**a**). Hollow ceramic artifacts can serve as thermometers for tea brewing, when hot water poured on the head of the zisha ceramic pee-pee boy (**b**) triggers a water jet. The jetting distance correlates with the water temperature. Frame rate is about 1 s. (Fig. 1(a) is not covered by the CC BY license. Frog-shaped whistle, 618–906. China. Porcelaneous ware, molded and with black glaze. Courtesy of Asian Art Museum of San Francisco, The Avery Brundage Collection, B69P33. Photograph © Asian Art Museum of San Francisco. All rights reserved, used with permission).

Then, the pee-pee boy is dried and placed on the vertical platform shown in Fig. 1, in preparation of the jetting. The wetting angle of water on the ceramic is 59 ± 4°, so that internal water tends to fill the orifice until the meniscus motion is stopped at the intersection of the orifice and the dried external wall, when Laplace pressure overcomes the static pressure. Then, water of the small beaker is brought to the desired temperature, measured with a thermocouple (uncertainty of ±2 °C), and poured onto the head of the pee-pee boy for a duration of 1–4 seconds, a time typically needed to trigger the jetting. The typical amount of water needed to produce a vigorous jet lasting several seconds is about 40 mL, a quantity equivalent of a small cup of tea. Paper towels on the horizontal platform absorb the water and facilitate the measurement of the jet length. A Reflex Digital camera (Canon EOS 70D) captures the jet trajectory as a movie, at a typical rate of 7 images per second. The initial slope θ of the trajectory of the water jet is measured from the movie. The typical jet trajectory is found to be a parabola. The temperature field and evolution of the pee-pee boy is measured with an infrared camera (FLIR A655sc, with an uncertainty of ± 2 °C).

### Model

As shown in Fig. 3, we assume that a thermodynamic process transforms the heat brought by the hot water into mechanical work –here the expansion of the internal air pocket that drives the jet, and into internal energy –here a general elevation of the pee-pee boy temperature.

To model this process, the following equations are written. A one-dimensional approximation of Fourier’s heat conduction law, modeled according to Ohm’s law, describes how much heat is transferred between the poured liquid and the ceramic wall, with respective indices L and 2


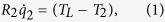


and between the wall and the internal air pocket, with index 1,


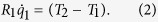


The resistances to heat transfer R_1_ and R_2_, shown in Fig. 3b, are obtained using conduction and convection heat transfer correlations^39^. Radiation heat transfer is neglected, which is a valid assumption for mixed convection-radiation problems at temperatures below 100 °C.


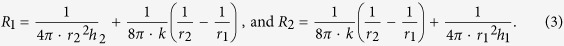


Above, the symbols r_2_, r_1_, h_2_, h_1_, and k stand respectively for the representative radius of the head, of the air pocket, the convection heat transfer coefficients between the ceramic and the outside fluid (water during the pour, then air), between the ceramic and the inside air, and the thermal conductivity of the ceramic. Equation (3) describes R_2_ as the sum of the resistance to heat transfer by convection between the surrounding fluid and the ceramic, and the resistance by conduction across the exterior half the ceramic wall. The resistance R_1_ sums the resistance by conduction across the interior half the ceramic wall and the resistance by convection between the ceramic and the air pocket.

The first principle of thermodynamics is then applied to the ceramic wall as


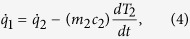


and to the air pocket as


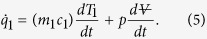


The above two energy balances express that heat provided by the poured water increases the internal energy of the wall and air, and in the latter equation, that the heated and expanding air pocket powers the jet. Here, p, 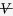, c_1_, and m_1_ are the internal pressure and air volume, the specific heat and mass of the air, respectively.

By combining the above heat transfer equations with the thermodynamic energy balances, the above equations become a system of two ordinary differential equations


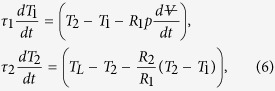


with time constants *τ*_1_ = (*mcR*)_1_, and *τ*_2_ = (*mcR*)_2_, where m_1_ and m_2_ are the respective mass of the air pocket and of the adjacent solid wall. Possibly, the internal water is heated in parallel to the internal air pocket. As shown in the supplementary documentation, the time constant of water heating is more than two orders of magnitude larger than that of the air, principally because of the larger heat capacity of the water. This causes the water to remain at room temperature, as indicated by the measurements in Fig. 4. Since room temperature is well below saturation temperature, the evaporation of the water is neglected, and the internal air pocket is considered dry.

The dynamics of the system is constrained as follows. First, the ideal gas law


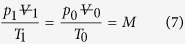


describes the expansion of the internal air pocket, where M is a constant and the atmospheric pressure and temperature are respectively p_0_ and T_0_. Then, neglecting the viscous and inertial losses in the narrow and short pee hole (where the flow is typically laminar), the pressure difference across the pee hole drives the water jet according to the Bernoulli equation, so that along the streamline 1-j in Fig. 3, we have


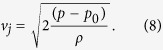


Here, *p*_*1*_ is the pressure of the air pocket, and the density of the water *ρ* = 1000 *kg*/*m*^3^. Interestingly, in the limit of inviscid flow, the maximum theoretical jetting velocity above is independent of the diameter of the pee hole. Finally, conservation of mass ensures that the rate of change of the air volume is proportional to the water jetting velocity *v*_*j*_


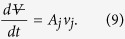


As Torricelli first reported in the 17^th^ century, the area A_j_ of the jet is typically smaller than the average measured hole area, by a factor *C* ≅ 0.5 for reentrant orifice as in the pee-pee boy. This phenomenon is due to inertial bending of the streamlines passing through the pee hole.

The above system of equations (6) to (9), 10, 10, 10 is a system of ordinary differential-algebraic equations for the five unknowns T_2_, T_1_, *p*_*1*_, v_j_, and 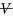. These equations can be solved numerically with an in-house advancing temporal scheme, or with a dedicated solver, such as *ode23* in the Matlab software, a fast, high-level, simple programming language^40^.

Once v_j_ is obtained, the jetting distance and trajectory can be modeled using ballistic equation (10)-an example is described in ref. 41. In the equations below, the following scalars are used: *g* is the acceleration of gravity, *θ* is the jetting angle, v_j_ is the initial velocity of the water jet, (*x*_0_, *y*_0_) are the initial positions of the jetting location, x and y are the respective horizontal and vertical locations of the jet trajectory.


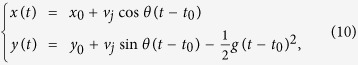



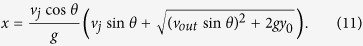


Combining both equations (10) when y(t) = 0 yields equation (11) for the horizontal jetting distance x measured on the floor. Note that these equations neglect air resistance. In that case, equation (11) simplifies into the well-know result of the maximum reach D of a parabolic trajectory initiated at *y*_0_ = 0,


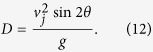


The following limiting case can be considered to describe the jetting capabilities of an optimally designed pee-pee boy: consider a pee-pee boy with an empty head, *infinitely larger* than his pee hole. In that case, the volume of the air cavity can be considered constant during the heating and jetting process. In that situation, the air pocket has time to reach the temperature of the hot poured water (a condition technically called thermal equilibrium), without significant expansion. In that *isochore* case, the pressure difference between the air pocket and the environment is maximum, so that the jet velocity no longer depends on the pee hole diameter, but simply on the ratio of temperatures of the hot fluid over the environmental temperature. Combining equations (7) and (8) for that isochore case gives


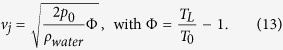


The dimensionless number Φ expresses the maximum thermodynamic driving force driving the jet.

## Results and Discussion

To guide the study described in this manuscript, the following hypotheses have been sequentially tested, that (i) *the jet is caused by thermal expansion of an internal air pocket;* (ii) *the model captures the physics and scales of the measurements*; (iii) *the pee-pee boy is a useful thermometer for tea brewing*; and (iv) *the pee-pee boy is well designed*. This section integrates results and their discussion.

**Figure 2:**
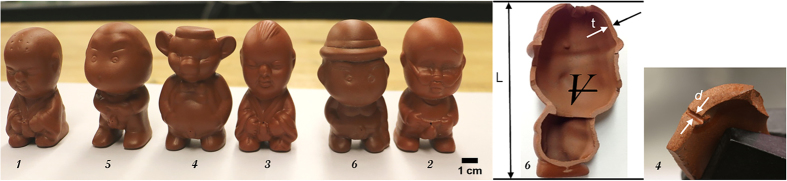
Six ceramic pee-pee boys line up before the experimental study. Static characterization reveals a hollow internal structure of artifact #6 and the pee hole of artifact #4. Numbers are for identification purpose.

To test hypothesis (i), we performed static measurements on six pee-pee boys, reported in Fig. 2 and Table S1. As detailed in the supplementary documentation, average height and head circumference were measured as 69 mm and 117 mm, respectively. The ratio of head circumference to height of 1.5 is disproportionate, about three times that of an average 5-year old, and five times that of an adult^42^,^43^. An average wall thickness *t* = 1.8 *mm* was used in simulations, unless specified.

Dynamic measurements of the jetting performance were performed, controlling the temperature and duration of the water poured on top of the pee-pee boy, measuring the mass m (empty, before and after jetting), the jet angle θ, the maximum jetting distance D, and the jetting duration τ_j_. All the measurements reported in Table S1 were made by pouring water at 100 °C. The resulting jetting distances ranged between 40 cm and 153 cm. From inspection of short-exposure pictures, the contraction of the jet *C* = 0.43 ± 0.05. The ambient temperature was measured as 23 ± 2 °C. The transient temperature field is shown with infrared measurements in Fig. 4; it indicates that, once the water pour stops (t > 7 s), the lower portion of the pee-pee boy is at a lower temperature than the upper portion of the head. This difference is a sign of the presence of air versus water: the ceramic-water system has a higher effusivity than the ceramic-air system^44^ and therefore absorbs more heat while increasing less in temperature.

**Figure 3:**
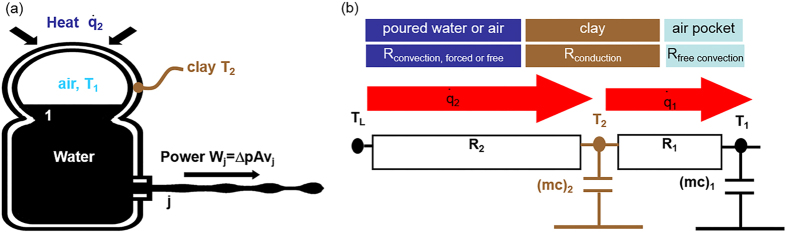
(**a**) Schematic representation of the pee-pee boy as a thermodynamic device converting some of the heat poured onto its head into the mechanical power of a water jet. In (**b**), the heat transfer equations are coupled with thermodynamic balance equations and fluid mechanics equations to produce the system of differential and algebraic equations (6, 7, 8, 9) which describes the dynamical jetting of the pee-pee boy.

**Figure 4:**
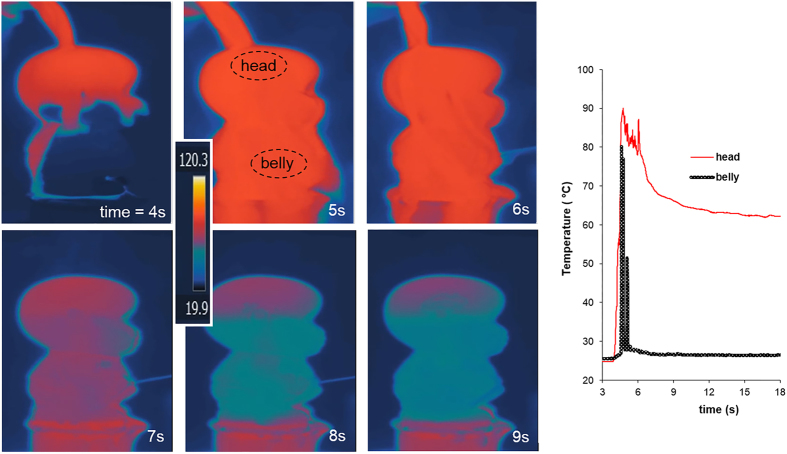
Infrared temperature measurements reveal the spatial distribution of temperatures on the left, and their transient evolution in jetting experiment with pee-pee boy #2 on the right. The strong difference in temperatures at the top of the head and in the belly at t ≅ 8 s indicates the presence of an internal air pocket in the head.

**Figure 5:**
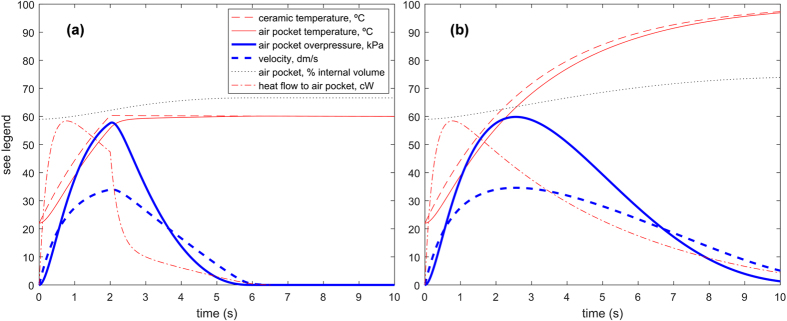
(**a,b**) The theoretical model described by equations (6, 7, 8, 9) shed light on the dynamical behavior of the pee-pee boy. Water at 100 °C is poured for 2 s (**a**) or 10 s (**b**). The temperatures, jet velocity, volume of the air pocket and heat flow received by the pocket are plotted as a function of time for pee-pee boy #2.

The remaining parameters needed for the simulations were obtained as follows. Zisha ceramic thermal conductivity has been measured^45^ as 1.3 W/m·K. Convection coefficients are estimated with standard heat transfer correlations^39^. Outside, the water-ceramic and air-ceramic convection coefficients are respectively equal to 8000 and 3, in W/m^2^K. Inside, the convection coefficient *h*_*1*_ depends on the size r_1_ of the air pocket as *h*_1_ = *Nu*·*k*_1_/*r*_1_, where *Nu* = 15^46^ and *k*_*1*_ are the respective Nusselt number quantifying internal convection and the thermal conductivity of the air. Typical values for pee-pee boy #2 of the thermal resistances shown in Fig. 3 are R_1_ = 9.4 Km^2^/W and R_2_ = 0.16 Km^2^/W (during the pour) and 61 Km^2^/W (after the pour), corresponding to time constants τ_1_ = 0.23 s and τ_2_ = 2.95 s (during the pour) and 1118 s (after the pour). Dynamic results of the thermodynamic model are presented in Fig. 5. Interpretation of those numbers tell us that during the pour, the thermal transport between the ceramic and the poured hot liquid controls the rate of heat transport. As a result, it takes a time corresponding to τ_2_, that is about 3 s, for the heat of the poured water to significantly change the internal air temperature (and pressure). The pouring of water should therefore last on the order of 3 s, a value comparable to the values of τ_p_ in Table S1. After the pour, little thermal transfer takes place between the pee-pee boy and the outside air, because of the very large value of τ_2_, as confirmed by the measurements in Fig. 4.

Predicted jetting distances in Table S1 are in very good agreement with the measurements, with a relative difference that does not exceed 30%. Predicted jetting durations are typically 30% to 70% longer than in the measurements. Possibly, this is due to a threshold amount of pressure or inertia needed to actually create and sustain a jet^47^, rather than water flowing down the legs of the tea pet. This threshold is not considered in the model, where the jetting duration is simply taken as the time while the velocity is non-zero.

Simulation results, like those in Fig. 5, shed light on the dynamic behavior of the device. The two plots show different situations for the same pee-pee boy #2 on which water at 100 °C is poured. In case (a), hot water is poured for 2 s, while in case (b) the pour lasts for the entire simulation time. In (a), the internal temperature is seen to increase throughout the duration of the pour, towards a maximum reached slightly later than the end of the pour. During the pour, the temperature of the internal air pocket increases towards that of the hot water, at a rate controlled by the convective and conductive thermal resistances and the heat capacity of the ceramic and air. The temperature, pressure, and volume of the air pocket increase monotonically, as well as the temperature of the ceramic. The heat flow into the air pocket reaches its maximum before the internal temperature peaks. When the pouring stops, the heat flux peaks and experiences a sharp and steep decrease. This behaviour seems simultaneous with that of the air pressure and temperature, because the time constant τ_1_ of the heat diffusion to the air pocket is much smaller than 1 s. The jetting velocity reaches its maximum at the same time as the internal pressure does, as dictated by equation (8). During the jetting, the temperature remains approximately constant because of the low convective heat transfer coefficient of the ambient air. On Fig. 5b, where the hot water is poured during the entire simulation time, the maxima of internal pressure and temperature, and of jetting velocity, occur later, at about 3 seconds, and reach higher levels than those of the case on the left. That time corresponds to the largest time constant of the system. The ceramic temperature values in Fig. 4 are about 10 °C higher than simulated in Fig. 5. The reason is that the IR measurement is at the surface of the ceramic and the temperature plotted in Fig. 5 is averaged over the thickness of the ceramic. Note that the maximum velocity reached in the (b) case, ~3.5 m/s is about half that of the theoretical isochore maximum of 7.4 m/s, predicted with equation (13). A reason is that the gas pocket expands at a rate that causes a non-negligible expansion before the temperature of the gas pocket reaches its maximum. In design terms, as will be discussed below in equation (19), this corresponds to a violation of the design condition that requires *π*_1,2_<<1.

Based on the agreement between simulations and experiments, as well as the direct measurements of the hollow geometry and the presence of an air pocket, it is reasonable to conclude that assumptions (i) and (ii) are valid, that is, the jet is caused by thermal expansion of an air pocket; and the model captures the physics and scales of the measurements.

**Figure 6:**
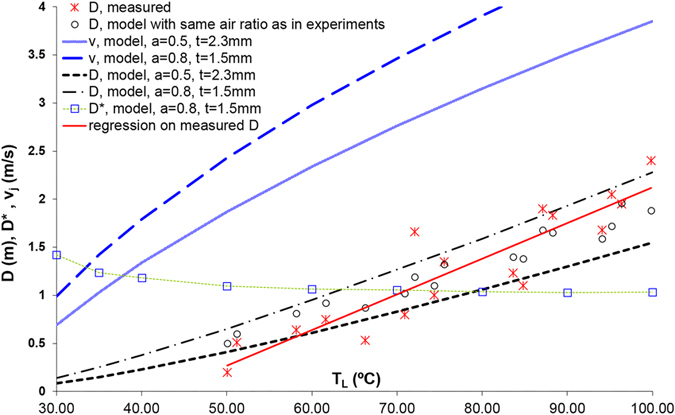
Characterization of pee-pee boy #1 as a temperature sensor. Measured and modeled jetting distances D, as well as predicted jetting velocities v_j_, are plotted as a function of the water temperature T_L_. The dimensionless jetting distance D* is given in equation (15), and *α* and *t* are respectively the air ratio and the wall thickness.

The discussion is now ripe to assess hypothesis (iii), that the pee-pee boy is a useful thermometer for tea brewing. Figure 6 describes how the jetting distance D of pee-pee boy #1 varies with the poured water temperature, for a pouring time of 4 s. The horizontal axis plots the water temperature. The air ratio in the experiments (crosses) was measured between 51% and 80%, and simulations (circles) were carried out with the same air ratio. Defining θ = T_L_ − T_0_, a linear fit of the experimental results shows that the sensitivity of the thermometer 
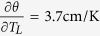
, while for the simulation 
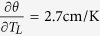
, ranging from 2.1 cm/K to 3 cm/K for the indicated range of combinations of wall thickness and air ratio. Using the Student t-distribution for finite sample size (here 17 measurements), the temperature uncertainty^48^ of a measurement with a calibrated pee-pee boy can be determined as


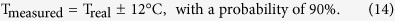


The above uncertainty probably results from the lack of repeatability in the process of pouring water on the device, and in the range of measured values of air ratio after filling the device, which vary between 50 and 80%. While both authors have little expertise in tea brewing, this level of accuracy is sufficient to discriminate between a water temperature at 100 °C versus 60 °C, the latter temperature corresponding to the extraction of only half the amount of antioxidants of green tea in comparison to the former^49^.

The above measurements of sensitivity and accuracy confirm that the pee-pee boy is an adequate temperature sensor for tea brewing, which validates assumption (iii). In technical terms, the pee-pee boy is a sensor and a transducer^50^, which transforms a thermal input signal (the water temperature) into a mechanical displacement output (the jet length). It is a *passive transducer* since it works without any inputs apart from its input signal, the water temperature, and the environment. Various kinds of sensors have various modes of providing output signals, e.g. optically, electrically, and mechanically. The pee-pee boy, in that context, is a mechanical sensor with the unique ability to produce a linear displacement up to 30 times larger than its own size. To the best of our knowledge, this is an outlier among typical displacement sensors such as rulers and caliper; angular sensors such as bubble levels; pressure sensors such as piezometer, manometers and Bourdon gauge spring-based force sensors; velocity sensors such as Pitot tubes; and temperature sensors such as bimorphs and liquid-in glass thermometers. All these but the pee-pee boy produce displacements of either equal dimension or smaller than their size. In that sense, the pee-pee boy is probably one of the few, if not the only, large-displacement mechanical sensor.

Figure 6 also shows D*, which is the measured jetting distance divided by the theoretical jetting distance for the case where the jet originates at ground level, i.e. y_0_ = 0).


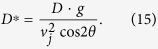


The fact that the numerical values of D* are larger than 1 at T < 65 °C simply means that the size of the pee-pee boy enhances the simulated jetting distance. The value of D* tends towards 1 for temperatures higher than 65 °C, showing that the height of the pee-pee boy offers little advantage for longer jets. In other words, being tall provides a negligible advantage in peeing the farthest.

One might wonder if the heat engine represented by the pee-pee boy could be used as a thermopneumatic pump, for instance in electronics or solar applications. To turn the one-shot process of the pee-pee boy into a cycle, it would require the additional steps of cooling down to ambient temperature with e.g. a bowl of cold water, and refilling. This could be done with e.g. the use of a check valve and a better connection to the environment temperature. The thermodynamic efficiency is however a typical bottleneck of thermopneumatic pumps. Thermodynamic efficiency is defined as the ratio of the mechanical power, i.e., the volume flow rate *Q* times the velocity *v*, over the heat flow 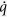. The efficiency 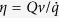 equals either 1.5 × 10^−8^ or 5 × 10^−10^, for the respective situations where the heat flow is accounted for as the heat transferred to the ceramic, or the enthalpy of the poured water. This efficiency is 7 to 9 orders of magnitude lower than the efficiency of a reversible thermodynamic cycle operating between the same temperature levels of T_L_ = 100 °C and T_0_ = 23 °C, 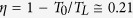. The efficiency is however comparable to the values of 10^−7^ to 10^−9^ reported in the review of micro-thermopneumatic pumps by Laser and Santiago^51^, which operate between two isochore and two isotherm curves, like a Stirling cycle^52^. In its present form, the proper technical name for the pee-pee boy is a single-action displacement (non-reciprocating) thermopneumatic pump^51^, an expression that lacks the evocative power of the pee-pee boy.

The last assumption (iv) to test is about the optimality of the design of the pee-pee boys. Let us first define a figure of merit, which would be the jetting velocity, which is theoretically proportional to the square root of the jetting distance, as per equation (15). From equation (13) we know that the jetting velocity increases monotonically with the thermodynamic driving potential Φ, and with the temperature T_L_ of the poured water. An optimum pee-pee boy would therefore simply be that with a jetting velocity equal to the thermodynamic maximum expressed in equation (13). That ratio is reached in the isochore heating case, where heating induces a maximum pressure build-up before any significant expansion by jetting reduces that pressure. Mathematically, the optimum isochore situation translates into


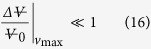


with 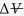 the jetted volume and 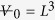 the volume of the air pocket. Considering 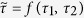 as a timescale representative of the combination of both timescales in equation (6), possibly their sum or average, the above criterion translates into


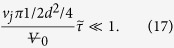


Using the thermodynamic definition of the velocity, equation (13), and lumping the numerical factors into the RHS, we obtain


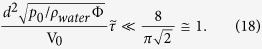


Criterion (18) can be expressed in turn with the two design limits where 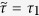 and 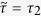 as defined below equation (6). Two dimensionless numbers π_1_ and π_2_ appear, and both need to be significantly smaller than one for the pee-pee boy to be optimally designed:


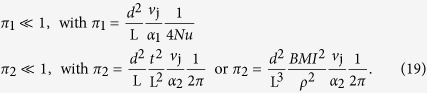


Each π compares two characteristic timescales, as explained in Table 1. Note that in the second definition of π_2_, we have used the body mass index (BMI, the ratio of dry mass over height square), which is easy for a shopper to test. Values of π_1_ and π_2_ are reported in Table S1. These, as well as other design constraints are summarized in Table 1.

Considering both criteria π_*1*_ ≪ *1* and π_*2*_ ≪ *1*, the size *L* should be as large as possible and the pee hole *d* should be as small as possible; the temperature difference should be as large as possible (to maximize v_j_), the thermal diffusivity of the material should be as high as possible. The criterion π_*2*_ ≪ *1*, adds the requirement that the shell or skin should be as thin as possible, or (equivalently) that the body mass index of the tea pet should be as small as possible. Interestingly, traditional fabrication of zisha pottery involves repeated beating of the clay until it becomes leather-hard and as thin as 2 or 3 mm. In fact, Geoffrey Gowlland mentioned in the personal communication that potter apprentices spend the first months, sometimes up to a year, beating the clay with a mallet-like tool into perfectly flat and even thin sheets and disks. The clay sheets are then warped around a mold made of wood, stone or plaster, then finally baked.

Additional criterion in Table 1 put 500 μm as a lower bound on the size of the pee hole, otherwise it would be impossible to fill the tea pet before the jetting experiment. Similarly, there is a lower bound of 300 μm on the thickness of the ceramic, based on the thinnest potteries on historical record. An upper bound on 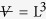 simply states that by their function, tea pets should have the typical size of a tea cup.

**Figure 7:**
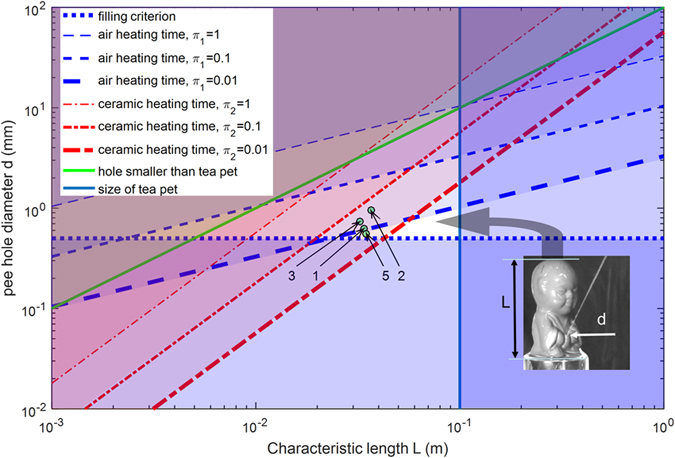
Graphical representation of the design space and rules of Table 1, in terms of two measurable variables, the diameter of the pee-hole and the size of the pee-pee boy. Colored areas violate at least one of the criteria of Table 1, delimited by lines of same color. The optimum design space, with jetting velocities reaching the thermodynamic maximum, is a white rhomboid. The numbers 1, 2, 3 and 5 represent the individual designs of pee-pee boys in Fig. 2, with L the cubic root of their volume.

A MATLAB script is used to simulate the thermofluidic behavior of a pee-pee boy using the criteria of Table 1, and the results are represented in the design space of Fig. 7, with respect to two directly measurable design parameters indicated in the inset picture, its size and the diameter of its boyhood. Colored regions in Fig. 7 designate regions where at least one criterion (that of the line with the same color) is violated. Three values of the heating time criteria (π_i_ = 1, 0.1, 0.01) are plotted for each π_1_ and π_2_, which according to numerical simulations correspond to jetting velocities at about respectively 10%, 50% and 90% of the maximum theoretical jetting velocity given by the thermodynamic limit (13). An arrow points to the optimum design space, a white rhomboidal region in which the jetting velocity is at least 90% of the theoretical maximum. The bold numbers 1, 2, 3 and 5 refer to the individual designs of pee-pee boys studied in this manuscript, which are in the region with velocities between 50 and 90% of the thermodynamic maximum, in agreement with results in Table S1. The last criterion that θ = 45° with respect to the horizontal, will maximize the jetting distance, for a given velocity.

For the craftsman, the above optimization discussion translates into molding pee-pee boys as large as customers are willing to buy, with the skin as thin, and the pee hole as small as possible. Zisha ceramic appears as the ceramic of choice for this endeavor, because of its high iron content thereby increasing the value of diffusivity α_2_ in equation (19). For the buyer, the criterion on π_2_ commands the selection of large and light tea pets.

From Fig. 6, two operating conditions emerge that maximize the jetting distance: (1) fill the pee-pee boy with as much air as possible, and (2) use the warmest water.

To summarize, the ideal pee-pee boy is tall with a large air-filled head, has a penis of the appropriate size and angle, and a thin and sensitive skin. Such tea pet would be a strong candidate for a “pissing contest”, a popular and primitive competition of ego among the males of the human^53^,^54^ and lobster^55^ species. Note that the latter are able of jets up to seven body lengths forward, just like the pee-pee boys studied here.

While this manuscript is mainly about the thermodynamics of the pee-pee boy, it leaves open the question as to when this ingenious and simple thermometer was invented. If the invention occurred earlier than the end of the 16th century, it would precede the 1592 invention of the thermoscope by Galileo Galilei, the first device in the Western world known to indicate temperature. Remarkably, the thermoscope was based on the thermally-driven expansion of an air pocket and its effect on the motion of an air-water interface^56^, similarly to the pee-pee boy. It is well known that potteries have been produced in China as far back as the Neolithic and that the presence of at least one hole is necessary in hollow potteries, to prevent explosive breakup driven by internal pressure during baking. While our review of books on zisha ceramic did not identify the period when pee-pee boys were invented, the whistle in Fig. 1a shows that hollow Chinese potteries have been produced as far back as the Tang Dynasty (618–906 CE).

## Conclusion

A thermodynamic analysis including experiments and modeling has been performed on six pee-pee boys made of zisha ceramic. The study demonstrates that the jet is driven by thermopneumatic action of an expanding internal air pocket. The jetting velocities and distance are found to be bounded by a thermodynamic maximum, a function of temperature. Seven design criteria to maximize the jetting distance are identified, including two dimensionless parameters. One dimensionless parameter is found to depend on the body mass index of the pee-pee boy. The sensitivity and uncertainty of the temperature measurement are quantified, and found adequate in the context of tea brewing.

## Additional Information

**How to cite this article**: Lee, V. and Attinger, D. Thermodynamics and historical relevance of a jetting thermometer made of Chinese zisha ceramic. *Sci. Rep*. **6**, 28609; doi: 10.1038/srep28609 (2016).

## Supplementary Material

Supplementary Information

## Figures and Tables

**Table 1:** The first six constraints and optimization rules aim at designing a pee-pee boy with jetting velocity approaching the thermodynamic maximum described in equation (13).

Design variable	Constraint	Physical meaning
Pee hole diameter d	d > 0.5 mm	Filling criterion. The Laplace pressure would prevent filling through smaller holes, by being larger than the pressure obtained when submerging the pee-pee boy under a few cm of water
Pee hole diameter d	d ≪ L	Hole smaller than the tea pet. This is an obvious geometric criterion.
Wall thickness	t > 0.3 mm	Minimum thickness of commercial ceramics, as in Roman and Chinese ceramics called eggshell porcelain^23^,^57^.
Volume of pee-pee boy	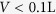	Aesthetics and water use commend that the tea pet is no larger than a cup of tea.
*π*_1_	*π*_1_ ≪ 1	Air heating time. π_1_ compares the time for heat to diffuse into the air-filled head with the time to change the volume of the air pocket by jetting.
*π*_2_	*π*_2_ ≪ 1	Ceramic heating time. π_2_ compares the time for heat to diffuse through the ceramic wall with the same time to change the volume of the gas pocket by jetting.
θ	θ = 45°	This angle maximizes the distance, as per equation (12).

The last constraint maximizes the jetting distance.
