# Supplementary material for: Thermodynamics and historical relevance of a jetting thermometer made of Chinese zisha ceramic
Source: Sci Rep. 2016 Jul 19;6:28609. doi: 10.1038/srep28609 (PMC4949419; doi:10.1038/srep28609)
Supplement: Supplementary Information [file srep28609-s1.doc]

**Supplementary documentation to: Thermodynamics and historical relevance of a jetting thermometer made of Chinese zisha ceramic**

**Vincent Lee^1,2^ and Daniel Attinger^1*^**

**^1^Mechanical Engineering Department**

**Iowa State University**

**Ames IA 50010, USA**

**^2^Mechanical Engineering Department**

**Georgia Institute of Technology**

**Atlanta, GA 30332**

***Corresponding author**

**Static characterization of the jetting thermometers:**

**To test hypothesis (i) that *the jet is caused by thermal expansion of an internal air pocket,* we performed static measurements on six pee-pee boys, reported in Figure 2 and Table S1. Typical height and head circumference were measured as 69mm and 117mm, respectively. The ratio of head circumference to height of 1.5 is disproportionate, about three times that of an average 5-year old, and five times that of an adult. The body mass index, BMI, the ratio of dry mass over height square, is also indicated in Table S1. For reference, the average BMI of a 5-year old boy is about 16, and the pee-pee boys, have a BMI of about one third or one half of that. Indeed pee-pee boys #3, 4 and 6, cut open, revealed a hollow inside, thin walls, and a single connection to their surroundings via the pee hole, as per Figure 2. The surface of the hole is observed to be smoother than that of the outer ceramic shell. Together with the protrusion towards the interior surface observed on the right inset of Figure 2, this hints that the pee holes might have been pierced from the outside of the unbaked ceramic with a needle-like object. The specific wall thickness *t*, averaged from about 10 locations each on pee-pee boys #3, 4 and 6, corresponds to an overall average of $t=1.8\text{mm}$ which was used in simulations, unless specified.**

| pee-pee boy # | L,  mm | P/L | m,  g | d,  µm | BMI,  kg/m^2^ | θ,  º | ,  cm^3^ | α,  % | t,  mm | τ_p_,  s | π_1_ | π_2_ | model  (%) | D, cm | | τ_j_,  s | |
| --- | --- | --- | --- | --- | --- | --- | --- | --- | --- | --- | --- | --- | --- | --- | --- | --- | --- |
|  |  |  |  |  |  |  |  |  |  |  |  |  |  | measured | model | measured | model |
| 1 | 66 | 1.5 | 31 | 624 | 7.2 | 50 | 38 | 36 | (1.8) | 4 | 0.06 | 0.11 | 52 | 153 | 152 | 6 | 8 |
| 2 | 72 | 1.5 | 35 | 943 | 6.8 | 15 | 49 | 59 | (1.8) | 2 | 0.10 | 0.11 | 47 | 70 | 80 | 4 | 6 |
| 3 | (69) |  | 28 | 731 | (5.9) | 20 | 33 | 18 | 1.55 | 3 | 0.10 | 0.25 | 63 | - | 63 | - | 3.5 |
| 4 | (69) |  | 33 | 571 | (6.8) | 0 | - | - | 1.65 |  |  |  |  | - | - | - | - |
| 5 | 69 | 1.9 | 36 | 558 | 7.6 | 0 | 42 | 59.0 | (1.8) | 3 | 0.037 | 0.047 | 55 | 60 | 55 | 10 | 14 |
| 5 | 69 |  | 36 | 558 | 7.6 | 0 | 42 | 36.1 | (1.8) | 3 | 0.043 | 0.076 | 61 | 40 | 51 | 6 | 10 |
| 6 | (69) |  | 35 | 625 | (7.3) | 0 | - | - | 2.26 |  |  |  |  | - | - | - | - |

**Table S1: Summary of static and dynamic measurements, compared to model results. Symbols L, P, m, d, BMI, θ, , α, t stand for the height, head circumference, empty ceramic mass, diameter of pee-hole, ratio of mass over height square i.e. body mass index, jet angle measured with respect to horizontal, internal volume, ratio of air volume to internal volume, and wall thickness. Symbols τ_p_, π_1_, π_2_,v_max_, v_ideal_, D, τ_j_, respectively stand for duration of the water pour (for a water temperature of 100ºC), the two dimensionless design parameters defined in equation (19), the maximum jet velocity during the experiment, the theoretical thermodynamic maximum velocity as per equation (13), the maximum jetting distance, and the duration of the jet. Values in parenthesis are average among several pee-pee boys.**

**Heating and evaporation of the water internal to the pee-pee boy:**

The thermodynamic model assumes that the water internal to the pee-pee boy remains at ambient temperature, with negligible evaporation and does not moisten the internal air pocket. A simple way to validate these assumptions is the inspection of the plot in figure 4, where IR measurements shows that the ceramic significantly heats up for the walls surrounding the top air pocket (head), and essentially remains at room temperature for the lower part of the pee-pee boy (belly) where the water is contained. Analytically, the following argument can be developed to estimate the heat transfer from the poured hot water to the water filling the tea pet. Using a one-dimensional approximation of Fourier’s heat conduction law, modeled according to Ohm’s law, the process of transferring heat to the water occurs in parallel to the heating of the internal air pocket described in Figure 3 of the manuscript.


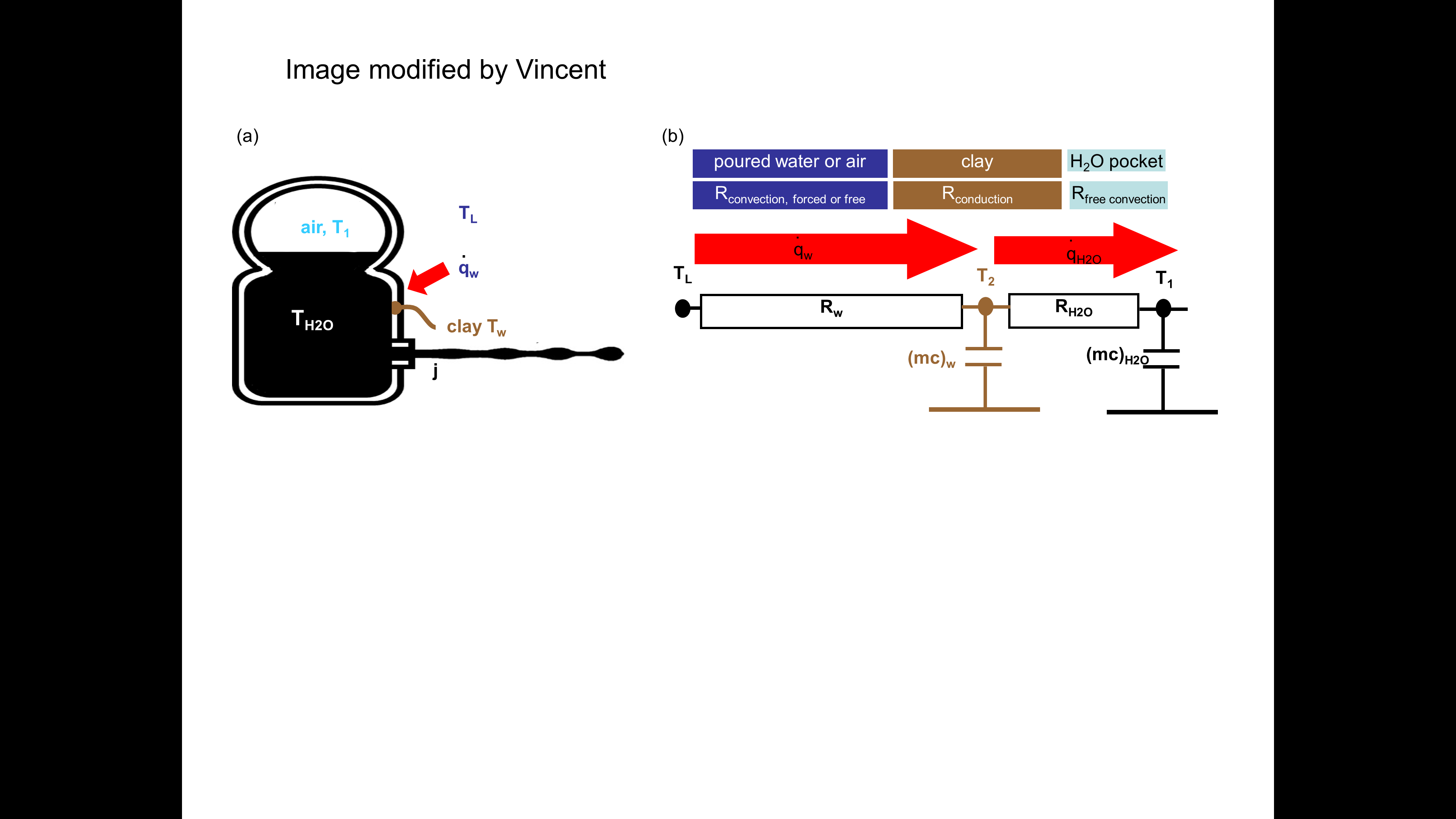


**Figure S1: (a) Schematic representation of the heat transfer from the poured water to the water contained in the belly of the pee-pee boy. In (b), the heat transfer equations are coupled with thermodynamic balance equations in an equivalent resistance network to write the equations below.**

Here, as shown in Figure S1, the heat transfer between the poured liquid and the ceramic wall, with respective indices L and w, follows the relation

| . | (1’) |
| --- | --- |

Similarly, between the wall and the water (with index H_2_O), we have

| . | (2’) |
| --- | --- |

The resistances to heat transfer R_w_ and R_H2O_, shown in Figure S1b, are obtained using conduction and convection heat transfer correlations[^3^](#_ENREF_3). Radiation heat transfer is neglected, which is a valid assumption for mixed convection-radiation problems at temperatures below 100ºC.

Considering that the volume of the liquid inside the tea pet is comparable to that of the air pocket, we have

| $R_{w}\simeq R_{2}$, and | (3’) |
| --- | --- |

Above, the symbols r_2_, r_1_, h_H2O_ and k stand respectively for the representative radius of the tea pet, of the water pocket, the convection heat transfer coefficient between the ceramic and the inside air, and the thermal conductivity of the ceramic. Resistance R_w_ is the sum of the resistance to heat transfer by convection between the surrounding fluid and the ceramic, and the resistance by conduction across the exterior half the ceramic wall. Equation (3’) describes the resistance R_H2O_ as the sum of the resistance by conduction across the interior half the ceramic wall and the resistance by convection between the ceramic and the internal water.

The first principle of thermodynamics is then applied to the ceramic wall as

| , | (4’) |
| --- | --- |

and to the internal water as

| . | (5’) |
| --- | --- |

The two above energy balances express that heat provided by the poured water increases the internal energy of the wall and of the energy pocket.

By combining the above heat transfer equations with the thermodynamic energy balances, the above equations become a system of two ordinary differential equations

|  | ,  , | (6’) |
| --- | --- | --- |

with time constants , and , where m_H2O_ and m_w_ are the respective mass of the internal water and of the adjacent solid wall.

Comparisons of equations 6’ with equations 6 in the manuscript shows that $\tau_{w}=\tau_{2}$. Calculations of the resistances to heat transfer can be done as follows. Convection coefficients are estimated with standards heat transfer correlations[^3^](#_ENREF_3). Outside, the water-ceramic and air-ceramic convection coefficients are respectively equal to 8000 and 3, in W/m^2^K. The convection coefficient $h_{H2O}=53$W/m^2^K between the internal water and the wall depends on the size r_H2O_ of the water pocket as , where $\text{Nu}=50$ and *k_H2O_*  are the respective Nusselt number[^4^](#_ENREF_4) quantifying internal convection and the thermal conductivity of the water. Typical values for pee-pee boy #2 of time constants τ_H2O_ = 522s and τ_w_= 3s (during the pour) and τ_air_ =0.3s. The finding that τ_H2O_ is two orders of magnitude larger than the duration of the pour indicates that the water can be considered isothermal, at room temperature, during the jetting process. Since room temperature is well below saturation temperature, the evaporation of the water and the moistening of the air are neglected.

**References**

1. Cole, T. J., Freeman, J. V. & Preece, M. A. British 1990 growth reference centiles for weight, height, body mass index and head circumference fitted by maximum penalized likelihood. *Stat. Med.* **17**, 407-429, doi:10.1002/(sici)1097-0258(19980228)17:4<407::aid-sim742>3.0.co;2-l (1998).

2. *WHO Child Growth Standards*. (World Health Organization, 2006).

3. Incropera, F. P. & DeWitt, D. P. *Fundamentals of Heat and Mass Transfer*. 3rd edn, (Wiley, 1995).

4. Duan, Y., Hosseinizadeh, S. F. & Khodadadi, J. M. Effects of Insulated and Isothermal Baffles on Pseudosteady-State Natural Convection Inside Spherical Containers. *J. Heat Transf.* **132**, 1-10, doi:10.1115/1.4000753 (2010).
